# 以白蛋白结合型紫杉醇为基础的方案治疗晚期肺癌的临床疗效及安全性观察

**DOI:** 10.3779/j.issn.1009-3419.2017.07.07

**Published:** 2017-07-20

**Authors:** 旭 李, 斌 艾, 萍 张, 琳 李, 晓楠 武

**Affiliations:** 100730 北京，北京医院肿瘤内科，国家老年医学中心 Department of Medical Oncology, Beijing Hospital, National Center of Gerontology, Beijing 100730, China

**Keywords:** 白蛋白结合型紫杉醇, 肺癌, 客观有效率, 疾病控制率, 不良反应, Albumin-bound paclitaxel, Lung neoplasms, Objective response rate, Disease control rate, Adverse events

## Abstract

**背景与目的:**

白蛋白结合型紫杉醇是通过将人血白蛋白与紫杉醇相结合的无需助溶剂的新型紫杉醇制剂，本研究旨在观察白蛋白结合型紫杉醇治疗晚期肺癌的临床疗效及其安全性。

**方法:**

选取2011年11月-2014年12月收治的进展期/不可手术的晚期肺癌患者共50例。给予白蛋白结合型紫杉醇130 mg/m^2^，第1天、第8天单药方案或者联合方案治疗，21 d为1个周期，观察每个周期不良反应，每2个周期按实体瘤疗效评价标准（Response Evaluation Criteria in Solid Tumors, RECIST）1.1进行影像学疗效评价。

**结果:**

白蛋白结合型紫杉醇治疗总体客观有效率（overall response rate, ORR）为20%，疾病控制率（disease control rate, DCR）为68%。在亚组分析中，鳞癌ORR为26.7%，DCR为80%，明显优于其他病理类型，但是尚未达到统计学差异。以白蛋白结合型紫杉醇单药或者两药联合的基础上联合抗血管生成治疗可以提高ORR（36.4% vs 15.4%）。四线及以上治疗患者DCR仍可达到69.2%。主要不良反应为血液学毒性但是可控制，无超敏反应及4级不良反应发生。

**结论:**

白蛋白结合型紫杉醇为基础的治疗方案治疗晚期肺癌无论其病理类型及治疗线数均有一定疗效，对于鳞癌及与抗血管靶向治疗联合时更有优势，即使对于老年及多线治疗后的患者耐受性较好。

## Abstract (en)

**Background and objective:**

Albumin-bound paclitaxel is a novel paclitaxel formulation formed by the combination of paclitaxel and human serum albumin (HSA) to improve the efficacy of paclitaxel and reduce its adverse reactions. The aim of this retrospective study is to observe the efficacy and safety of albumin-bound paclitaxel-based therapy in the treatment of lung cancer.

**Methods:**

We have enrolled 50 patients with advanced or unresectable retreatment lung cancer who were admitted from November 2011 to December 2014. All patients were treated with albumin-bound paclitaxel-based therapy with a 21 day cycle (albumin-bound paclitaxel weekly by intravenous dose of 130 mg/m^2^ on day 1 and day 8). Efficacy was evaluated every two cycles according to Response Evaluation Criteria in Solid Tumors (RECIST) 1.1 and side effects were observed during each cycle. All patients were evaluated.

**Results:**

The total objective response rate (ORR) of albumin-bound paclitaxel-based therapy was 20%, disease control rate (DCR) was 68%. In the subgroup analysis, in squamous non-small-cell lung carcinoma groups, ORR and DCR were 26.7% and 80% respectively. Albumin-bound paclitaxel based chemotherapy combined anti-angiogenesis therapy had a promising overall response rate 36.4%. In the patients who had been previously treated with≥4 lines of chemotherapy DCR also reached 69.2%. The most common adverse reactions were hematologic toxicities and were all manageable, no hypersensitivity reactions or treatment-related grade 4 adverse events were reported.

**Conclusion:**

Weekly albumin-bound paclitaxel is effective and well tolerated in the treatment of advanced lung cancer including all histological subtypes. Albumin-bound paclitaxel was associated with superior efficacy in patients with squamous (SCC) histology and combined with anti-angiogenesis therapy. Albumin-bound paclitaxel shown to be an effective and safe regimen for elderly or previous heavily treated patients.

在世界范围内肺癌是癌症死亡的主要原因，特别是对于男性患者^[1]^。肺癌中85%为非小细胞肺癌（non-small cell lung cancer, NSCLC），而其中40%诊断时即为转移性病变^[2, 3]^，5年生存率不超过5%^[4]^。紫杉醇是一种广谱的抗肿瘤药物常用于肺癌、乳腺癌等多种实体瘤的治疗。130 nm白蛋白结合型紫杉醇（nab-Pacilitaxel, Abraxane）是一种新型的紫杉醇制剂，无需蓖麻油作为助溶剂，因此可以减少传统的紫杉醇常见的毒性反应，同时其特殊结构可以通过内源性白蛋白通路使得更多的紫杉醇进入到肿瘤组织从而发挥抗肿瘤作用^[5, 6]^。Ⅲ期临床研究显示其临床疗效明显优于传统的紫杉醇制剂^[7]^，先后于2012年被美国食品和药物管理局（Food and Drug Administration, FDA）、2015年被欧洲批准用于NSCLC的治疗。同时也有相关研究^[8]^报道白蛋白结合型紫杉醇单药即使对于多线治疗后的小细胞肺癌也有一定的临床疗效。本研究对50例晚期肺癌无论其病理类型使用以白蛋白结合型紫杉醇为基础的治疗方案的临床病例进行回顾及分析，以探讨白蛋白结合型紫杉醇在治疗中的疗效及不良反应。

## 1 资料与方法

### 1.1 病例选择及一般资料

回顾性分析2011年11月-2014年12月北京医院收治的50例晚期肺癌患者的临床资料。纳入标准：经病理组织学证实为进展期或确诊无法手术的Ⅲ期/Ⅳ期肺癌。50例患者中男39例，女11例。年龄26岁-83岁，中位年龄62岁，60岁及以上35例（70%），65岁及以上19例（38%），70岁及以上9例（18%），80岁及以上3例（6%）。其中NSCLC 40例，腺癌25例，鳞癌15例；小细胞肺癌10例。诊断时参照1982年美国东部肿瘤协作组（Eastern Cooperative Oncology Group, ECOG）评分标准：0分-1分35例，2分15例。Ⅲ期5例，Ⅳ期45例，其中单器官/部位转移8例（占16%），2个器官/部位转移20例（40%），侵及≥3个器官/部位者17例（占34%）。其中化疗一线治疗8例（占16%），二线治疗16例（占32%），3线治疗13例（占26%），4线治疗8例（占16%），5线治疗4例（占8%），6线治疗1例（占12%）。

### 1.2 治疗方案

所有患者使用的白蛋白结合型紫杉醇（abraxane，美国新基药业有限公司）130 mg/m^2^，第1、8天（21 d为1个化疗周期），用药前不予抗过敏预处理，每次静脉滴注30 min，化疗期间观察并记录患者的不良反应。具体治疗方案如下：①白蛋白结合型紫杉醇单药治疗40例（80%）；②白蛋白结合型紫杉醇双药治疗方案10例（20%）；联合顺铂6例，联合卡铂2例，联合TS-1 1例，联合伊立替康1例；③50例中白蛋白结合型紫杉醇联合抗血管生成靶向治疗11例（占22%），肺腺癌占8例，鳞癌2例，小细胞癌1例；其中联合重组人血管内皮抑制素治疗7例，联合贝伐珠单抗4例，上述靶向药物均按药品说明书指示用药。全部患者均未预防性使用粒细胞集落刺激因子类药物（granulocyte colony stimulating factor, G-CSF），但出现3度以上的血液学毒性时则使用G-CSF治疗。治疗直至出现疾病进展及患者拒绝化疗或无法耐受化疗。

### 1.3 疗效、不良反应评价及随访

治疗前所有患者进行影像学基线检查并对所有可测量病灶进行基线测量并记录，每2个周期治疗后进行影像学评估。按照实体瘤疗效评价标准（Response Evaluation Criteria in Solid Tumors, RECIST）1.1进行评价：分为完全缓解（complete response, CR）；部分缓解（partial response, PR）；疾病进展（progressive disease, PD）；疾病稳定（stable disease, SD)。客观缓解率（objective response rate, ORR）=CR+PR，疾病控制率（disease control rate, DCR）=CR+PR+SD；无进展生存（progression-free survival, PFS）定义为开始治疗至第一次发生PD或任何原因死亡的时间间隔。不良反应按美国国立癌症研究所不良反应事件通用术语标准（National Cancer Institue-Common Terminology Criteria for Adverse Events, NCI-CTC）4.0判定，标准分为0级-4级。50例患者无失访，随访率100%。

### 1.4 统计学方法

采用SPSS 20.0（IBM Corp）进行统计学分析。临床疗效及不良反应等直接根据原始数据进行计算。临床特征与治疗疗效的比较采用χ^2^检验或*Fisher*精确检验，*P*＜0.05为差异有统计学意义。

## 2 结果

### 2.1 临床疗效

50例患者共完成142个周期的化疗（治疗周期数1个-6个），平均化疗周期数为2.84个。50例患者可评价疗效，未见CR患者，10例（20%）PR，24例（48%）SD，16例（32%）PD，ORR为20%（10/50），DCR为68%（34/50）。50例患者按照不同病理类型进行亚组分析，25例腺癌中5例（20%）PR，12例（48%）SD，8例（32%）PD，ORR为20%（5/25），DCR为68%（17/25）；15例鳞癌中4例（26.7%）PR，8例（53.3%）SD，3例（20%）PD，ORR为26.7%（4/15），DCR为80%（12/15）；10例小细胞肺癌中1例（10%）PR，4例（40%）SD，5例（50%）PD，ORR为10%（1/10），DCR为50%（5/10）。按治疗方案不同进行亚组分析，化疗组6例（15.4%）PR，20例（51.3%）SD，13例（33.3%）PD，ORR为15.4%（6/39），DCR为66.7%（26/39）；化疗联合靶向治疗组4例（36.4%）PR，4例（36.4%）SD，3例（27.2%）PD，ORR为36.4%（4/11），DCR为72.2%（8/11），接受化疗联合重组人血管内皮抑制素治疗的患者中有4例腺癌（3例SD，1例PD），2例鳞癌（1例PR，1例SD）和1例小细胞肺癌（PD），接受化疗联合贝伐珠单抗治疗的4例患者均为腺癌（3例PR，1例PD）。一线使用含白蛋白结合型紫杉醇方案治疗（*n*=8）的ORR为50%（4/8），DCR为62.5%（5/8），二线及以上使用（*n*=42）ORR为14.3%（6/42），DCR为69.0%（29/42）。

### 2.2 不良反应

74%的患者完成了2周期以上的治疗（治疗周期数：1个-6个），没有患者因为不良反应中断治疗且无治疗相关死亡的发生。以白蛋白结合型紫杉醇为基础的治疗方案常见的不良反应为血液学毒性，主要是白细胞降低[46% (23/50)]、中性粒细胞降低[36% (18/50)]和贫血[40% (20/50)]，但是大多数不良反应为1级-2级，2例出现3级白细胞降低，1例出现3级中性粒细胞降低。非血液学毒性主要表现为外周神经性麻木[38% (19/50)]、疲劳[42% (21/50)]、脱发[62% (31/50)]和胃肠道反应[56% (28/50)]等，均表现为1级-2级（表 1）。50例患者使用含白蛋白紫杉醇治疗方案整体耐受性良好且不良反应可控制，使用过程中也无过敏反应及超敏反应的发生。

**1:** 含白蛋白结合型紫杉醇方案治疗50例晚期肺癌的不良反应[*n*（%）] Percentage of patients with treatment-related toxicities/adverse events (*n*=50) [*n* (%)]

Adverse events	All grade	Grade 1	Grade 2	Grade 3	Grade 4
Hematologic					
Leukopenia	23 (46)	14 (28)	7 (14)	2 (4)	0
Neutropenia	18 (36)	12 (24)	5 (10)	1 (2)	0
Thrombocytopenia	8 (16)	6 (12)	2 (4)	0	0
Anemia	20 (40)	17 (34)	3 (6)	0	0
Non-hematologic					
Peripheral neurotoxicity	19 (38)	15 (30)	4 (8)	0	0
Fatigue	21 (42)	14 (28)	5 (10)	2 (4)	0
Alopecia	31 (62)	28 (56)	3 (6)	0	0
Arthralgia/Myalgia	9 (18)	7 (14)	2 (4)	0	0
Digestive tract reactions	28 (56)	22 (44)	6 (12)	0	0
Mucositis	6 (12)	5 (10)	1 (2)	0	0
ALT/AST elevation	7 (14)	6 (12)	1 (2)	0	0

在血液学毒性方面，可以观察到≥65岁患者不良反应发生率高于65岁以下患者，尤其贫血的发生率有显著性差异（57.9% *vs* 29%，*P*=0.043，表 2）。按治疗线数进行分组，不同治疗线数的患者血液学毒性的发生率无统计学差异。同时我们还可以观察到ECOG体能状态（performance status, PS）评分≥2分的患者血液学毒性的发生率明显高于低于2分的患者，并且有统计学差异（白细胞减少：66.7% *vs* 37.1%，*P*=0.055；中性粒细胞减少：60% *vs* 25.7%，*P*=0.021；贫血：66.7% *vs* 28.6%，*P*=0.012，表 2）。虽然联合治疗组血液学毒性的发生率也高于单药治疗组，但无统计学意义。

**2:** 按年龄、治疗线数、治疗方案及ECOG PS评分分组比较不良反应的发生率 Incidence of adverse events according to age, treatment line, therapeutic regimen and ECOG PS

Item	Age		*P*	Treatment line		*P*	Monotherapy/combination chemotherapy		*P*	ECOG PS		*P*
	< 65 (*n*=31)	≥65 (*n*=19)		1/2 line (*n*=24)	≥3 line (*n*=26)		Monotherapy (*n*=40)	Combination (*n*=10)		0-1 (*n*=35)	≥2 (*n*=15)	
Hematologic												
Leukopenia	13 (41.9)	10 (52.6)	0.461	11 (45.8)	12 (46.2)	0.982	17 (42.5)	6 (60.0)	0.523	13 (37.1)	10 (66.7)	0.055
Neutropenia	11 (35.5)	7 (36.8)	0.923	8 (33.3)	10 (38.5)	0.706	13 (32.5)	5 (50.0)	0.507	9 (25.7)	9 (60.0)	0.021
Thrombocytopenia	4 (12.9)	4 (21.1)	0.715	3 (12.5)	5 (19.2)	0.793	4 (10.0)	4 (40.0)	0.067	3 (8.6)	5 (33.3)	0.077
Anemia	9 (29.0)	11 (57.9)	0.043	8 (33.3)	12 (46.2)	0.355	15 (37.5)	5 (50.0)	0.718	10 (28.6)	10 (66.7)	0.012
Non-hematologic												
Peripheral neurotoxicity	12 (38.7)	7(36.8)	0.895	9 (37.5)	10 (38.5)	0.944	14 (35.0)	5 (50.0)	0.61	12 (34.3)	7 (46.7)	0.409
Fatigue	10 (32.3)	11 (57.9)	0.075	10 (41.7)	11 (42.3)	0.963	16 (40.0)	5 (50.0)	1	11 (31.4)	10 (66.7)	0.021
Alopecia	20 (64.5)	11 (57.9)	0.64	15 (62.5)	16 (61.5)	0.944	25 (62.5)	6 (60.0)	1	22 (62.9)	9 (60.0)	0.849
Arthralgia/Myalgia	6 (19.4)	3 (15.8)	1	4 (16.7)	5 (19.2)	1	7 (17.5)	2 (20.0)	1	5 (14.3)	4 (26.7)	0.52
Digestive tract reactions	16 (51.6)	12 (63.1)	0.425	13 (54.2)	15 (57.7)	0.802	19 (47.5)	9 (90.0)	0.039	16 (45.7)	12 (80.0)	0.025
Mucositis	3 (9.7)	3 (15.8)	0.844	2 (8.3)	4 (15.4)	0.741	4 (10.0)	2 (20.0)	0.744	3 (8.6)	3 (20.0)	0.506
ALT/AST elevation	2 (6.5)	5 (26.3)	0.122	1 (4.2)	6 (23.1)	0.129	4 (10.0)	3 (30.0)	0.262	3 (8.6)	4 (26.7)	0.213
ECOG PS: Eastern Cooperative Oncology Group performance status.												

在非血液学毒性方面，无论年龄是否≥65岁以及不同的治疗线数，不良反应的发生率均无显著性差异。而联合药物治疗组胃肠道反应的发生率明显高于白蛋白紫杉醇单药治疗组（90% *vs* 47.5%, *P*=0.039）；ECOG PS评分≥2分的患者疲劳（66.7% *vs* 31.4%, *P*=0.021）及胃肠道反应的发生率显著高于0分-1分的患者（80% *vs* 45.7%，*P*=0.025，表 3），且差异有统计学意义。

## 3 讨论

肺癌是一个组织学多样性的疾病，目前分为NSCLC和小细胞肺癌，前者约占肺癌总体的85%-90%；NSCLC又分为鳞癌和非鳞癌2种亚型；非鳞癌又包括腺癌，大细胞癌和其他非特异病理类型。进展期NSCLC一线化疗的标准方案为含铂二联方案，其中包括3代新药（如吉西他滨、紫杉醇等），而进展期SCLC一线标准化疗方案是依托泊苷联合铂的二联方案。130 nm白蛋白结合型紫杉醇（Abraxane, Celgene Corporation）是一种无需助溶剂的新型紫杉醇制剂，Ⅲ期研究显示与传统的紫杉醇制剂比较可以明显提高ORR，无论其组织病理类型还是年龄，也是一线进展期NSCLC的治疗选择之一^[7]^。国内外一些回顾性分析显示白蛋白结合型紫杉醇二线治疗NSCLC ORR可以达到20%-30%，明显高于公认的标准二线治疗药物多烯紫杉醇^[9-11]^，即使对于多线治疗的患者也可以达到18.75%的疗效^[12]^。一项来自日本的研究还显示对于二线治疗失败的难治或复发的小细胞肺癌，应用白蛋白紫杉醇单药治疗ORR达到了33%^[8]^。本回顾性研究进一步探索了以白蛋白结合型紫杉醇为基础的方案治疗晚期肺癌的疗效及安全性，并且根据临床特征进行相关分析以探索获益人群特征。

本研究结果显示在晚期肺癌的治疗中以白蛋白结合型紫杉醇为基础的治疗方案无论病理类型及治疗线数总ORR可以达20%，DCR达68%。而在肺癌的不同病理类型中，ORR和DCR均在鳞癌中最高，小细胞肺癌中最低，分别为26.7% *vs* 10%（*P*=0.61）、80% *vs* 50%（*P*=0.255），但是尚未达到统计学差异。国外的Ⅲ期研究对比白蛋白结合型紫杉醇与传统紫杉醇在治疗不同病理类型肺癌中的作用结果显示，在鳞癌一线治疗中ORR可以达到41% *vs* 24%（*P*＜0.01），并且mOS也有延长的趋势（10.7 mo *vs* 9.5 mo）（*P*=0.28）^[9, 13]^，本研究再次证明了对于肺鳞癌的患者应用以白蛋白结合型紫杉醇为基础的治疗方案可以取得更好的ORR和DCR，对于小细胞肺癌仍有一定的临床活性。本研究还显示在以白蛋白结合型紫杉醇单药或者两药联合的基础上联合抗血管生成治疗可以较单纯化疗取得更好的ORR（36.4% *vs* 15.4%）（*P*=0.267）。一项来自美国的研究也提示在白蛋白结合型紫杉醇及卡铂的基础上联合贝伐珠单抗治疗进展期NSCLC的6个月PFS率可以达到74%（95%CI: 57-97），ORR达36%，中位至疾病进展时间（median time to progression, mTTP）8.5个月以及mOS 12.2个月^[14]^。本研究所用的抗血管生成治疗包括了贝伐珠单抗和重组人血管内皮抑制素两种不同的药物，化疗联合抗血管生成治疗组ORR高于单纯化疗组，并且ORR与上述国外的研究结果相似，因此也提示我们在以白蛋白结合型紫杉醇的化疗基础上联合抗血管生成治疗也是晚期肺癌治疗的理想选择之一。通过本研究结果我们还发现随着治疗线数的增加，ORR逐渐下降（*P*=0.122），对于四线及以上治疗的患者其ORR只有7.7%，但是DCR始终可以维持在60%-80%，即使是四线及以上治疗DCR仍可达到69.2%，并且不同线数治疗DCR无显著差异（*P*=0.84），因此对于多线治疗的晚期肺癌患者我们仍可以考虑选择应用白蛋白结合型紫杉醇。

在治疗相关不良反应中没有4级毒性反应的发生，上述患者用药过程中也未发生过敏反应及超敏反应，未发生治疗相关毒性反应导致的治疗延迟、中断或死亡。最常见的不良反应包括白细胞减低、中性粒细胞减低、贫血以及脱发、疲劳、胃肠道反应和外周神经麻木。3级以上的不良反应有白细胞减低，中性粒细胞减低以及疲劳。对于老年患者（≥65岁）仅贫血的发生率较高并且有统计学差异，相对比较安全；而ECOG PS评分≥2分的患者血液学毒性以及疲劳和胃肠道反应的发生率相对比较显著，因此在临床应用时要特别注意，剂量可以根据个人情况进行适当调整。在非血液学毒性方面，外周神经病变是紫杉类药物治疗中最常见的不良反应。在Ⅲ期临床研究中白蛋白结合型紫杉醇的外周神经病变明显低于传统的紫杉醇制剂（46% *vs* 62%, *P*＜0.001）^[7]^。本研究中外周神经麻木的发生率38%，也明显低于传统的紫杉醇制剂，且无3级/4级不良反应的发生，再一次显示出白蛋白结合型紫杉醇的优势。总体来说白蛋白结合型紫杉醇130 mg/m^2^第1天、第8天单药方案或者与其他化疗药联合的方案不良反应相对较轻并且可控制，即使是对于老年或者多线治疗后的患者耐受性仍较好。国内外的相关研究探索白蛋白结合型紫杉醇的最佳剂量与给药方式，结果显示130 mg/m^2^，第1、8天（21 d为1个化疗周期）的给药方式较260 mg/m^2^每3周1次的给药方式具有更佳的疗效及耐受性，并且明显降低了外周神经病变及肌肉关节疼痛的发生，具有更好的临床获益-风险比，特别是对于老年患者^[15-17]^。鉴于我们收治的患者大部分为老年、复治的患者，考虑到患者的耐受性，因此采用了130 mg/m^2^，第1、8天（21 d为1个化疗周期）的给药方式。我们的研究结果再次证实了这种给药方式不仅可以获得满意的ORR及DCR，同时极少出现3级/4级不良反应，安全性良好。

本研究作为回顾性分析存在一定的局限性，首先入选的患者例数相对有限，不同病理类型的患者例数也不均衡；其次患者接受的治疗方案不统一，治疗线数涵盖了从一线到六线治疗的患者，因此临床疗效的可比性及不良反应评估的意义有所欠缺。但是本回顾性研究显示了以白蛋白结合型紫杉醇为基础的方案对于晚期肺癌无论年龄以及初治或者复治安全性较好；在肺癌的不同病理类型中鳞癌的ORR、DCR最有优势，但是对于小细胞肺癌同样有效，即使是二线及以上治疗的患者DCR仍可接近70%。以白蛋白结合型紫杉醇为基础的化疗方案如果能联合抗血管生成治疗将会取得更好的ORR，上述结果对临床工作均具有一定的参考价值。为了进一步增加结果的说服力，未来还需扩大研究病例数以进行更好的研究分析。
